# Supplementary material for: Adapting the informed push model to the last mile of the contraceptive supply chain in South Kivu in the Democratic Republic of Congo
Source: PLOS Glob Public Health. 2024 Aug 8;4(8):e0003531. doi: 10.1371/journal.pgph.0003531 (PMC11309402; doi:10.1371/journal.pgph.0003531)

PLOS Global Public Health

Adapting the informed push model to the last mile of the contraceptive supply chain in the Democratic Republic of Congo: Pilot study results

--Manuscript Draft--

**Manuscript Number:**	PGPH-D-23-01903
**Article Type:**	Research Article
**Full Title:**	Adapting the informed push model to the last mile of the contraceptive supply chain in the Democratic Republic of Congo: Pilot study results
**Short Title:**	Adapting the informed push model to the contraceptive supply chain in the DRC: Pilot Study
**Corresponding Author:**	Katherine H LaNasa<br>Tulane University School of Public Health and Tropical Medicine<br>New Orleans, LA UNITED STATES
**Order of Authors:**	Katherine H LaNasa
	Michel Yalaza
	Felix Hitayezu
	Frank Roijmans
**Keywords:**	Contraception; Family Planning; Supply Chain Management; Stockouts
**Abstract:**	In the Democratic Republic of Congo (DRC), contraceptive security is one of the largest barriers to improving access to family planning. This article presents findings from a pilot study which adapted an informed push distribution model to the last mile of the contraceptive supply chain, between the health zone pharmacy and health facilities, in the eastern region of DRC. The pilot intervention consisted of three changes in the supply chain: organization of most efficient transportation routes, in depth involvement of the community in supply chain management and bundling of contraceptives with other essential medications for delivery. The pilot was implemented from October 2017 to October 2018 in the Katana health zone of the South Kivu province. Although there was not a statistically significant difference in availability of contraceptives during the pilot study period, there were sharp declines in the mean length of stockouts at health facilities and the monthly transportation costs of delivering contraceptives. Based on lessons learned from the pilot study, including the essential involvement of community members in the model, the intervention was revised to cover larger geographic areas while maintaining an efficient flow of contraceptives and renamed the Comité Axes de Distribution (CAD). Overall, the pilot study demonstrated it is feasible to adapt the IPM to a new location with complex geographical, political and socioeconomical influences. Future studies will be required to rigorously evaluate the revised model to determine whether the CAD is more effective than the existing pull supply chain system for contraceptives in the DRC.
**Additional Information:**	
**Question**	**Response**
**Financial Disclosure**<br><br>Enter a financial disclosure statement that describes the sources of funding for the work included in this submission and the role the funder(s) played. This includes grants and any commercial funding of the work or authors.	Funding for this project was provided by the Royal Netherlands Embassy as part of the Commodities, Chain and Care Project, led by UNFPA DRC. The supply chain component of the project for this study was executed by i+Solutions. The funders had no role in study design, data collection and analysis, decision to publish, or preparation of the manuscript.

Powered by Editorial Manager® and ProduXion Manager® from Aries Systems Corporation

This statement will be typeset if the manuscript is accepted for publication.<br><br>*Please review the submission guidelines and the instructions link below for detailed requirements and guidance.*	
**Competing Interests**<br><br>On behalf of all authors, disclose any competing interests that could be perceived to bias this work.<br><br>This statement will be typeset if the manuscript is accepted for publication.<br><br>*Please review the instructions link below and PLOS Global Public Health's competing interests policy to determine what information must be disclosed at submission.*	Katherine H LaNasa received funding from i+ solutions as an academic writing consultant to assist in preparing this manuscript.
**Data Availability**<br><br>Before publication, Authors are required to make fully available and without restriction all data underlying their findings. Please see our PLOS Data Policy page for detailed information on this policy.<br><br>A **Data Availability Statement**, detailing where the data can be accessed, is required at first submission. Insert your Data Availability Statement in the box below.<br><br>Please see the data reporting section of our submission guidelines for instructions on what you need to include in your Data Availability Statement.<br><br>This statement will be typeset if the	Data is available via Zenodo at 10.5281/zenodo.8367959

manuscript is accepted for publication.<br><br>*PLOS allows rare exemptions to address legal and ethical concerns. If you have legal or ethical restrictions, please use the box below to detail these in full sentences for the Journal team to consider.*	

Adapting the informed push model to the last mile of the contraceptive supply chain in the Democratic Republic of Congo: Pilot study results

Katherine H LaNasa[1*], Michel Yalaza[2¶], Felix Hitayezu[3¶], Frank Roijmans[4]

Author affiliations

[1] Deparment of Health Policy and Management, School of Public Health and Tropical Medicine, Tulane University, New Orleans, Louisiana

[2] i+solutions, Kinshasa, DRC

[3] i+solutions, Kigali, Rwanda

[4] i+solutions, Woerden, Netherlands

* Corresponding author

E-mail: kschulze@tulane.edu

¶ These authors contributed equally to this work.

Abstract

In the Democratic Republic of Congo (DRC), contraceptive security is one of the largest barriers to improving access to family planning. This article presents findings from a pilot study
that (not which)
which adapted an informed push distribution model to the last mile of the contraceptive supply chain, between the health zone pharmacy and health facilities, in the eastern region of DRC. The
more
pilot intervention consisted of three changes in the supply chain: organization of most efficient
in-depth
transportation routes, in depth involvement of the community in supply chain management and bundling of contraceptives with other essential medications for delivery. The pilot was implemented from October 2017 to October 2018 in the Katana health zone of the South Kivu province. Although there was not a statistically significant difference in availability of contraceptives during the pilot study period, there were sharp declines in the mean length of stockouts at health facilities and the monthly transportation costs of delivering contraceptives. Based on lessons learned from the pilot study, including the essential involvement of community members in the model, the intervention was revised to cover larger geographic areas while maintaining an efficient flow of contraceptives and renamed the Comité Axes de Distribution (CAD). Overall, the pilot study demonstrated it is feasible to adapt the IPM to a new location with complex geographical, political and socioeconomical influences. Future studies will be required to rigorously evaluate the revised model to determine whether the CAD is more effective than the existing pull supply chain system for contraceptives in the DRC.

Introduction

The Democratic Republic of Congo (DRC) ranks 179th out of 186 countries on human development indicators according to the United Nations[1], reflecting a low life expectancy, educational index and standard of living. The DRC has the third largest population (86,790,000) in Sub-Saharan Africa (SSA)[2] and the third highest fertility rate in the region[3]. Although the DRC government has shown support for family planning (FP) by establishing the first national strategic plan for family planning in 2014[4], and revised again in 2022[5] the modern contraceptive prevalence rate (mCPR) among all women has remained low (16.3%), while nearly a third of women (31.8%) have an unmet need for FP[6].

third-largest
third-highest

Increasing women's reliable access to contraceptives is a key component to addressing unmet need for FP[7] and increasing mCPR[8]. This requires a functioning supply chain to deliver the right type of contraceptives, in the right amount, and at the right time to service providers[9], and is one of the six pillars in the WHO framework for strengthening health systems[10]. Typically, the final section of the supply chain between the health zone storage and the service provider, commonly referred to as the last mile, is the weakest link in the system[11].

sentence needs work; perhaps break into two sentences
link or component?

The contraceptive supply environment in the DRC is particularly challenging. As the largest country in SSA in terms of land mass, the vast size and poor transportation infrastructure pose massive barriers for distribution of health products. Additionally, the government has neither the financial nor human capital to invest in a nationwide well-functioning supply chain. As a result, the current contraceptive supply chain is a weak piecemeal system dependent on the patchwork support of international donors[9]. In the last mile of the supply chain in the DRC, health products are traditionally distributed using a pull system where lower-level health facilities order commodities as needed. This system places a financial burden on health

in which

facilities[12], requires already overworked health care providers to spend time on logistics rather than providing services to clients[13, 14], and is dependent on accurate and timely forecasting to predict what commodities will be needed, which can be challenging in low-resource settings where training is limited[13, 15]. The 2018 Service Provision Assessment (SPA) indicated that only half of the health facilities in the DRC that provide FP services had contraceptive methods in stock and available for clients on the day of the survey[16], indicating the supply chain is failing and Congolese women's access to contraception is threatened[17, 18].

A relatively new supply chain model, termed the Informed Push Distribution Model (IPM), has shown promising results for strengthening the last mile of the contraceptive supply chain in several other countries in SSA[19, 20]. The first version of the IPM was introduced to the international FP field in Zimbabwe, where dedicated delivery truck teams were used to "top-up" health facilities' contraceptive stocks on a fixed schedule[19]. In the next version of the model, implemented in Senegal, a dedicated logistics manager was added to the system to improve stock reporting and help deliver contraceptives[20]. Following the success of the Senegal IPM, which demonstrated significant reductions in stockouts and improved communication throughout the logistics system[20, 21], versions of the IPM have been adapted to a handful of other contexts, including the Direct Deliver and Information Capture system in Nigeria[22, 23], the Dedicated Logistics Systems in Mozambique[24], and the IPM in Togo[25] with largely positive results. However, the model is highly influenced by the geography of each location; regional road infrastructure can affect the mode of commodity transportation and delivery time, and weather patterns (rainy or dry seasons) can make travel especially difficult in rural areas[22, 23]. In addition, differences in resources, such as financial support and internet access, and varying levels of political support for FP may require adapting implementation

"may require" or "require"? Your next sentence said that adaptaion is required.

strategies of the IPM to each new context. Therefore, given the variability in environments and the strong interest in strengthening FP supply chains across SSA, it is important to test the IPM in a variety of contexts and adapt the model as needed.

The objective of this study was then to demonstrate the feasibility and acceptability of implementing an adapted version of the IPM in the South Kivu province of the DRC to strengthen the last mile of the contraceptive supply chain. Located in the Eastern region of the country, South Kivu is characterized as a largely rural province with significantly weak road infrastructure and limited internet access. The province also faces high security risks from ongoing internal conflicts, as well as the recent and prolonged 2018 outbreak of Ebola in the region. The project began by piloting the model in one health zone in South Kivu, with the goal of using the lessons learned to further adapt and scale-up the model to more areas in the province or country.

Methods

Pilot intervention

suggest "improve delivery in the last mile of the..."

The pilot IPM was adapted to the DRC context to change the last mile of the contraceptive supply chain in health zones (HZ) which typically operated using a pull system that required the head nurse at each health facility to manage stock reporting, submit order requisitions to HZ management, and coordinate transportation of the requested commodities from the central HZ pharmacy to the health facility. The adapted pilot model, termed the

changes to more or "the most"

Community-IPM (C-IPM), consisted of three changes in the supply chain: organization of most efficient transportation routes, involvement of community members in the supply chain management, and bundling of contraceptives with other essential medications for delivery.

To adapt the C-IPM to the geographic environment, first the existing infrastructure and spatial distribution of health facilities was assessed to design optimal transportation routes from the central HZ pharmacy to facilities, resulting in five transportation routes, termed distribution axes in the model (Fig 1). For each axis in the model, a community member was appointed as a C-IPM focal point. Focal points were trained to work with the head nurse at each health facility along their assigned distribution axis to collect reports of monthly commodity consumption and order requisitions from health facilities, and jointly with the HZ authorities to arrange for the delivery of products. Due to limited internet access in the HZ, all stock reports and orders were prepared on paper. An agreed upon monthly reporting date was determined for each axis on which the focal point would begin at the furthest health facility along the axis and drive up the route collecting reports until reaching the HZ pharmacy. Then, the focal point would hire a local private motor taxi to pick up the requested contraceptives and drive back down the distribution axis to resupply the health facilities along the way. The decision to involve community members as logistic focal points in the pilot C-IPM was aimed at adapting the model to a low-resource setting to strengthen sustainability of the model by encouraging local buy-in to the project. The C-IPM focal points were not paid directly for their involvement in the pilot; however, they received a per diem and costs were covered for transportation.

Figure 1. The informed push distribution model (IPM) implemented in the last mile of the contraceptive supply chain of the DRC. IPM focal points were each assigned a distribution axis to deliver contraceptive products from the central health zone pharmacy to the health facilities along the axis.

Pilot implementation & assessment

The pilot C-IPM was tested in the Katana health zone (HZ) of the South Kivu province of the DRC. Katana is a rural HZ, approximately 45 km from the nearest city of Bukavu, which contains 20 health facilities that serve a population of 236,986. The local geography is mountainous, and the road infrastructure is unpaved. The area is inhabited mainly by the Bashi tribe and residents are predominately Christian.

To evaluate the pilot IPM, contraceptive commodities and stock reports from the health facilities were collected by the IPM focal points throughout the study period. The intervention recorded the availability of nine contraceptive methods: male condoms, female condoms, oral pills (Microglyn and Microlut), injectables (Depo-Provera), IUD, implants (Jadelle and Implanon NXT) and emergency contraceptive pills. Availability of all contraceptive products at the health facilities was assessed at the beginning and end of the study period with a health facility assessment that recorded whether each method was in stock and not expired on the day of the survey. A stockout was defined as one or more methods of contraception unavailable or expired on the day of the survey. Monthly reports were submitted by the focal points to track the duration of contraceptive stockouts, delivery time of products and transportation costs. The timeliness and completeness of submitted monthly stock reports were also assessed.

Changes

Change in key pilot study indicators between baseline and endline facility assessments was compared using chi-squared tests. Data were analyzed using STATA 17.0 (Stata Corp, College Station, Texas, US). Given the small sample size of health facilities, significance was assessed at the 0.10 level.

Ethics statement

Ethical approval for this study was obtained from the Institutional Review Board of Tulane University, which determined the pilot evaluation did not constitute human subjects research as defined by the Common Federal Rule and thus review was not required (Study Reference #: 2023-1533).

Pilot Results

Availability of contraceptives increased among the 20 health facilities in Katana during the study period (Table 1). At baseline, 65% of facilities had all nine contraceptive methods in stock, while at endline this proportion had increased to 75%. The proportion of reported submitted on time increased from 75% to 80%, while the completeness of reports slightly decreased from 85% to 80%. However, the change in these two indicators was not statistically significant between baseline and endline surveys. The mean delivery time of products was also similar between baseline (1.6 days) and endline (1.7 days). However, there was a sharp decline in the mean duration of stockouts over the study period, from 7-12 days during the first two months of the study to 0-1 days throughout final 10 months of the study (Fig 2)

Table 1. Change in key indicators between baseline and endline surveys

	Baseline n=20	Endline n=20	p-value
All methods available at survey (%)	65.0	75.0	0.49
Reports submitted on time (%)	75.0	80.0	0.71
Reports completed (%)	85.0	80.0	0.68
Delivery time in days (mean)	1.6	1.7	0.39

Figure 2. Mean length of contraceptive stockouts at health facilities in the Katana health zone of South Kivu, DRC.

Four months into the project, health facility staff and HZ management requested that other essential medications for the treatment of malaria, tuberculosis and HIV be delivered together with the contraceptive supplies to maximize transportation efficiency and minimize total delivery costs of other health products in the HZ. This request demonstrated that local health officials found the new supply chain model acceptable and suggests they were invested in sustaining the design beyond the study period. This request was also met with success as the delivery costs for contraception markedly declined by the third month of the project, from $20 US to $5 US or less, while the delivery cost for other essential medications also declined from $45 US at the beginning of the program to $2 US by the end of the study period (Fig 3).

Figure 3. Monthly transportation costs of delivery contraceptives and other essential medications through the adapted IPM to health facilities in the Katana health zone of South Kivu, DRC.

Overall, the pilot intervention demonstrated it was feasible to implement an adapted version of the IPM to the challenging context of a rural HZ in the DRC. Further, the intervention was met with a high level of acceptability from community members, health facility staff and HZ officials.

Lessons Learned

One key component to adapting and implementing the pilot IPM was the involvement of community members in the program. First, it was beneficial to work within the existing health sentence is confusing zone management structure and members of the community, and later by involving the CODESA "for" (instead of "to") to appoint community members as focal points. This process enhanced community buy-in to the intervention quickly and may improve sustainability of the model beyond international donor funding. Additionally, bundling contraceptives with other essential medications streamlined the delivery process and lowered transportation costs for these medications in the HZ, which further strengthened health officials' support for the model. This process compares to the IPM implemented in Senegal which also began bundling contraceptives and essential medications at the insistence of regional health managers[20]. Finally, for the supply chain to function properly, it is essential that stock reports and contraceptive order requests are complete and turned in on time so the contraceptive needs for the HZ and province can be accurately forecasted and procured. Future IPM designs may consider implementing an incentive system to ensure prompt reporting and encourage increased ownership of the system by focal points.

Revised Intervention Design

Given the encouraging results of the pilot study, there was interest from health officials in South Kivu to expand the intervention to other rural HZs. However, some HZs in the province cover a very large geographic area with dispersed health facilities, which the focal points could not reach in one day along their distribution axis. To adjust for the varying geography and size of HZs, the model was further revised and named *Comité Axes de Distribution* (CAD). In this revised model, a health facility located near the middle of the distribution was identified and were then transport branches then were organized like spokes around the facility (Fig 4). Community members in the HZ were elected as focal points for the CAD by the existing HZ management

structure, the *Comité de Développement Sanitaire* (Sanitary Development Committee), or CODESA, which is a community-led committee that manages the primary health care facilities and leads sanitary development in the HZ. The focal point was responsible for picking-up the bundled contraceptives and essential medications from the central health zone pharmacy as usual, but then delivered the products to the identified central health facility on their distribution axis. Then, other appointed focal points picked-up the supplies and transported them to the remaining health facilities along their transport branch. The goal of the revised CAD is to efficiently cover a large geographic area in a short amount of time to keep the flow of contraceptive products moving on a reliable and timely schedule.

suggest "health development," as the word "sanitary" in English doesn't work well here

no hyphen ("picking up")

Figure 4. Revised version of the adapted IPM, termed CAD, in the South Kivu province of the DRC. Each distribution axis is divided into additional transport branches to efficiently cover a large geographic area in a short amount of time.

Discussion

This study is the first to implement an adapted version of informed push distribution model (IPM) in the DRC, first termed the Community-IPM and later revised to *Comité Axes de Distribution* (CAD). The flexible design of the revised CAD allows this intervention to be implemented in a wide variety of environments, which is essential in a country as large as the DRC with an array of geographies where one size will not fit all. However, there are some limitations to the intervention approach and pilot study. First, there may have been other factors influencing the contraceptive supply chain during the pilot study period. A nurses' strike took place between May and August of 2018, during which stock reports were not submitted on time

and supplies were not delivered to facilities resulting in stockouts of multiple contraceptive methods. Further, activities from other FP organizations working in the area may have brought an influx of contraceptives at different times. Given the difficulty in coordination between FP projects in the area, which became apparent during the pilot study period, a contraceptive logistic group was added to the local Permanent Multisectoral Technical Committee (CTMP), a group of key stakeholders which guides FP initiatives in the provinces. Further, the pilot C-IPM was only tested in one rural HZ and the revised CAD model has not been assessed yet. As the revised CAD model spreads to other HZs in South Kivu or even to other provinces, it will be important to rigorously evaluate the model using an experimental study design to determine whether the model significantly reduces stockouts and improves contraceptive availability compared to the existing pull supply chain system in the DRC.

Overall, given the large land area of the DRC and poor national infrastructure, building a strong supply chain is challenging, however, the pilot results indicate it is feasible to adapt the IPM to a new location with complex geographical, political and socioeconomical influences. Further, the revised CAD was designed based on lessons learned from the pilot study to better adapt the IPM to fit the supply environment of large rural areas with the goal of reliably delivering contraceptives through the last mile to health facilities and their clients.

Acknowledgements

The authors wish to thank Jane Bertrand who provided valuable guidance in development and editing of the manuscript. Furthermore, the authors would like to thank consultant Freddy Salumu for his involvement in the design of the study, the presentation of the study protocol to health authorities and the implementation and initial monitoring of the pilot and consultant

Gilbert Mungungu Witakenge for concluding the pilot and contributing to the final report. The authors also thank consultants Eugene Barhukenge and Justin Rukika, for their contributions in collecting and compiling data from the HZs, and the executives of South-Kivu DPS, Dr Zozo Musafiri and Dr Socrate Cuma for their contribution in the review of the methodology and the validation of the preliminary results. A special thanks to all HZ managers and FOSA service providers who took part in this study and the heads of the partner organizations who encouraged us and without whom this study would not have seen the light of day: Dr Arsene Binanga (Tulane University), Dr Dotian Wanogo (UNFPA), Dr Seydou Ndiay (Cordaid DRC) and Ph. Franck Biayi (PNAM/ MoH DRC). The Authors thank the Ministry of Foreign Affairs of the Netherlands and the Netherlands embassies in Rwanda and DRC who enabled realization of this study by funding the Jeune S3 project through Cordaid Netherlands, and the 3C project through UNFPA DRC.

References

1. Democratic Republic of the Congo: Human Development Indicators: United Nations Developmen Programme; [October 04, 2020]. Available from: http://hdr.undp.org/en/countries/profiles/COD.

2. Country profile: Democratic Republic of Congo [July 10, 2020]. Available from: http://data.un.org/en/iso/cd.html.

3. Sub Saharan Africa; Democratic Republic of Congo: World Bank; [August 23, 2020]. Available from: https://data.worldbank.org/indicator/SP.DYN.TFRT.IN?locations=ZG-CD&most_recent_value_desc=true.

4. Planification Familiale: Plan Stratégique National à Vision Multisectorielle (2014–2020). Kinshasa: MSP: Ministère de la Santé Publique (MSP), 2014.

5. Plan stratégique national a vision multisectorielle de la planification familiale 2021-2025 avec regard sur 2030. Kinshasa: MSP: Ministère de la Santé Publique (MSP), 2022.

6. FP 2030: FP Indicator Data File. In: Track20, editor. 2023.

7. Mukasa B, Ali M, Farron M, Van de Weerdt R. Contraception supply chain challenges: a review of evidence from low- and middle-income countries. Eur J Contracept Reprod Health Care. 2017;22(5):384-90. Epub 2017/10/31. doi: 10.1080/13625187.2017.1394453. PubMed PMID: 29087737.

8. Wang W, Wang S, Pullum T, Ametepi P. How Family Planning Supply and the Service Environment Affect Contraceptive Use: Findings from Four East African Countries. Calverton, MD: USAID, 2012.

9. Kwete D, Binanga A, Mukaba T, Nemuandjare T, Mbadu MF, Kyungu MT, et al. Family Planning in the Democratic Republic of the Congo: Encouraging Momentum, Formidable Challenges. Glob Health Sci Pract. 2018;6(1):40-54. Epub 2018/03/30. doi: 10.9745/GHSP-D-17-00346. PubMed PMID: 29602865; PubMed Central PMCID: PMCPMC5878077.

10. Everybody's Business: Strengthening Health Systems to Improve Health Outcomes: WHO's Framework for Action. Geneva: WHO: World Health Organization (WHO), 2007.

11. UNFPA Supplies Annual Report 2019. New York: UNFPA: UNFPA, 2020.

12. Hasselback L, Dicko M, Viadro C, Ndour S, Ndao O, Wesson J. Understanding and addressing contraceptive stockouts to increase family planning access and uptake in Senegal. BMC Health Serv Res. 2017;17(1):373. doi: 10.1186/s12913-017-2316-y.

13. USAID | DELIVER PROJECT TO. The Logistics Handbook: A Practical Guide for the Supply Chain Management of Health Commodities. Arlington, VA: 2011.

14. Using Last Mile Distribution to Increase Access to Health Commodities. Arlington, VA: USAID | DELIVER PROJECT, Task Order 4, 2011.

15. Yadav P. TH, Babaley M. Storage and Supply Chain Management. Geneva: World Health Organization, 2011.

16. Ecole de Santé Publique de Kishasa ESPK/Kinshasa RDdC, ICF. Republique Democratique du Congo Évaluation des Presentations des Services de souns de Santé EPSS 2017-2018. Kinshasa, République Démocratique du Congo: ESPK and ICF, 2019.

17. Farmer DB, Berman L, Ryan G, Habumugisha L, Basinga P, Nutt C, et al. Motivations and Constraints to Family Planning: A Qualitative Study in Rwanda's Southern Kayonza District. Glob Health Sci Pract. 2015;3(2):242-54. Epub 2015/05/13. doi: 10.9745/GHSP-D-14-00198. PubMed PMID: 26085021; PubMed Central PMCID: PMCPMC4476862.

18. Ross J, Stover J. Use of modern contraception increases when more methods become available: analysis of evidence from 1982–2009. Global Health: Science and Practice. 2013;1(2):203-12. doi: https://doi.org/10.9745/GHSP-D-13-00010.

19. USAID DELIVER PROJECT TO. Delivery team topping up: bringing about reliable distribution in difficult environments. Washington (DC): John Snow Inc, 2008.

20. Daff BM, Seck C, Belkhayat H, Sutton P. Informed push distribution of contraceptives in Senegal reduces stockouts and improves quality of family planning services. Glob Health Sci Pract. 2014;2(2):245-52. Epub 2014/05/13. doi: 10.9745/GHSP-D-13-00171. PubMed PMID: 25276582; PubMed Central PMCID: PMCPMC4168620.

21. Hasselback L, Gueye, B., Ndao, O., Ndour, S. K., & Cissé, C. Incentivizing access to family planning in Senegal via the informed push model. Journal of Pharmaceutical Policy and Practice. 2014;7(1):1-.

22. PROJECT UD. USAID | DELIVER PROJECT Final Country Report, Nigeria. Arlington, VA: USAID | DELIVER PROJECT, Task Order 4 and Task Order 7. , 2016.

23. USAID | DELIVER PROJECT TOa. Nigeria: Direct Delivery and Information Capture Activities: March 2012–August 2014. Abuja, Nigeria: USAID | DELIVER PROJECT, Task Orders 4 and 7, 2014.

24. Prosser W, Jaillard, P., Assy, E., Brown, S. T., Matsinhe, G., Dekoun, M., & Lee, B. Y. System redesign of the immunization supply chain: Experiences from Benin and Mozambique. Vaccine. 2017;35(17):2162-6.

25. Van de Weerdt R, editor Informed Push Model in Togo: A Best Practice in Public/Private Partnership for Supply Chain Excellence. Reproductive Health Supplies Coalition; 2015; Mexico: UNFPA.

Figure 1

Click here to access/download;Figure;Fig 1.tif

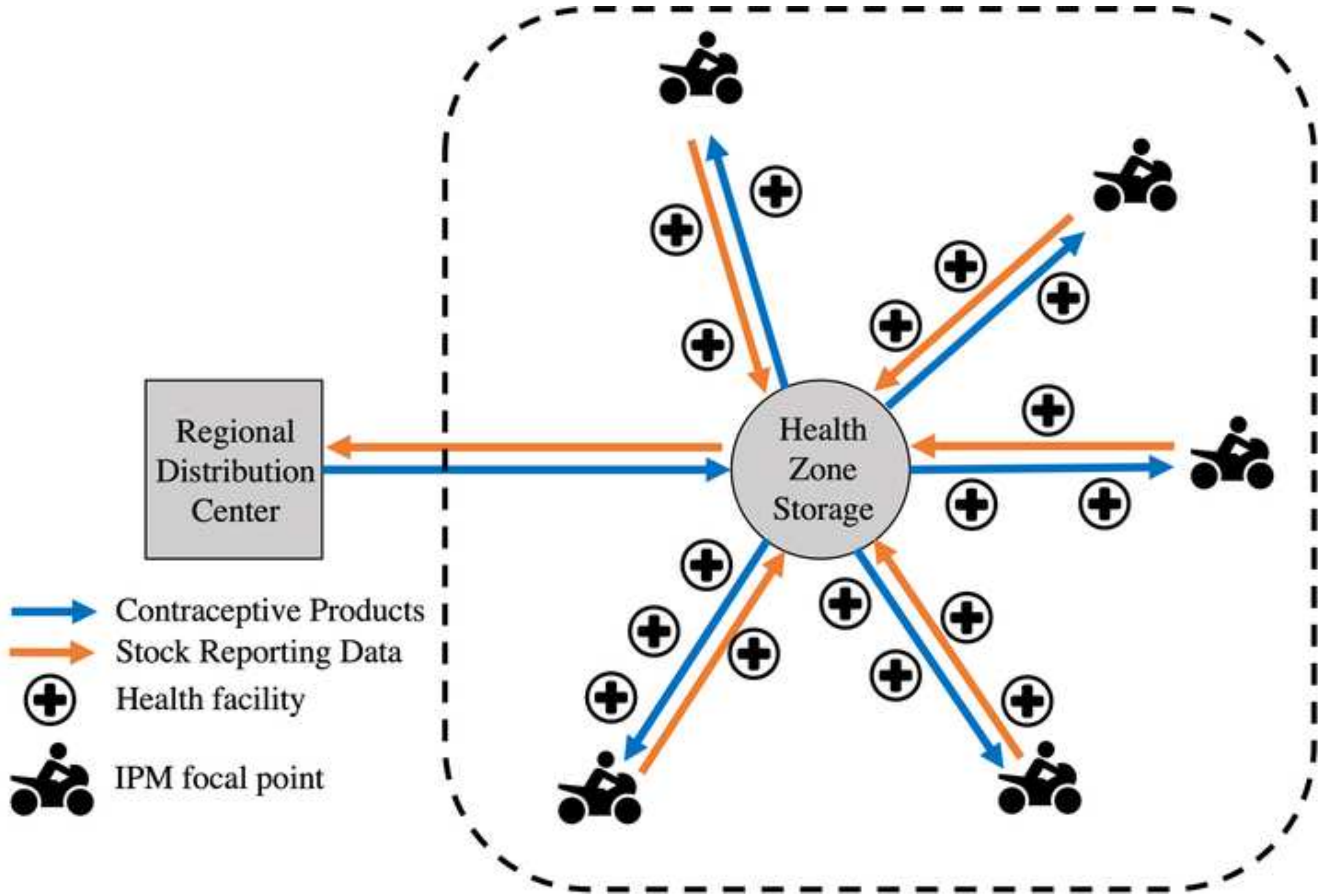

Figure 2

Click here to access/download;Figure;Fig 2.tif

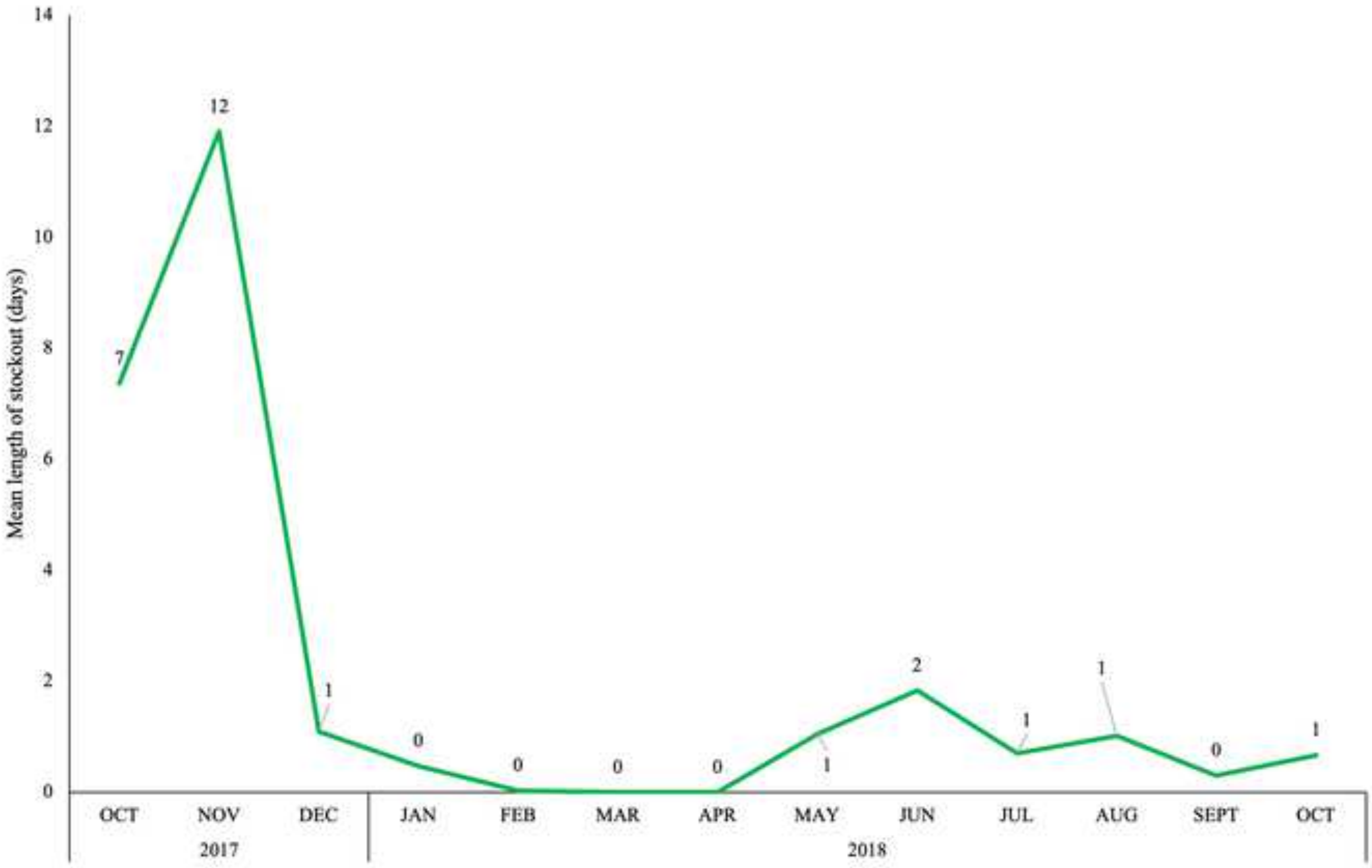

Figure 3

Click here to access/download;Figure;Fig 3.tif

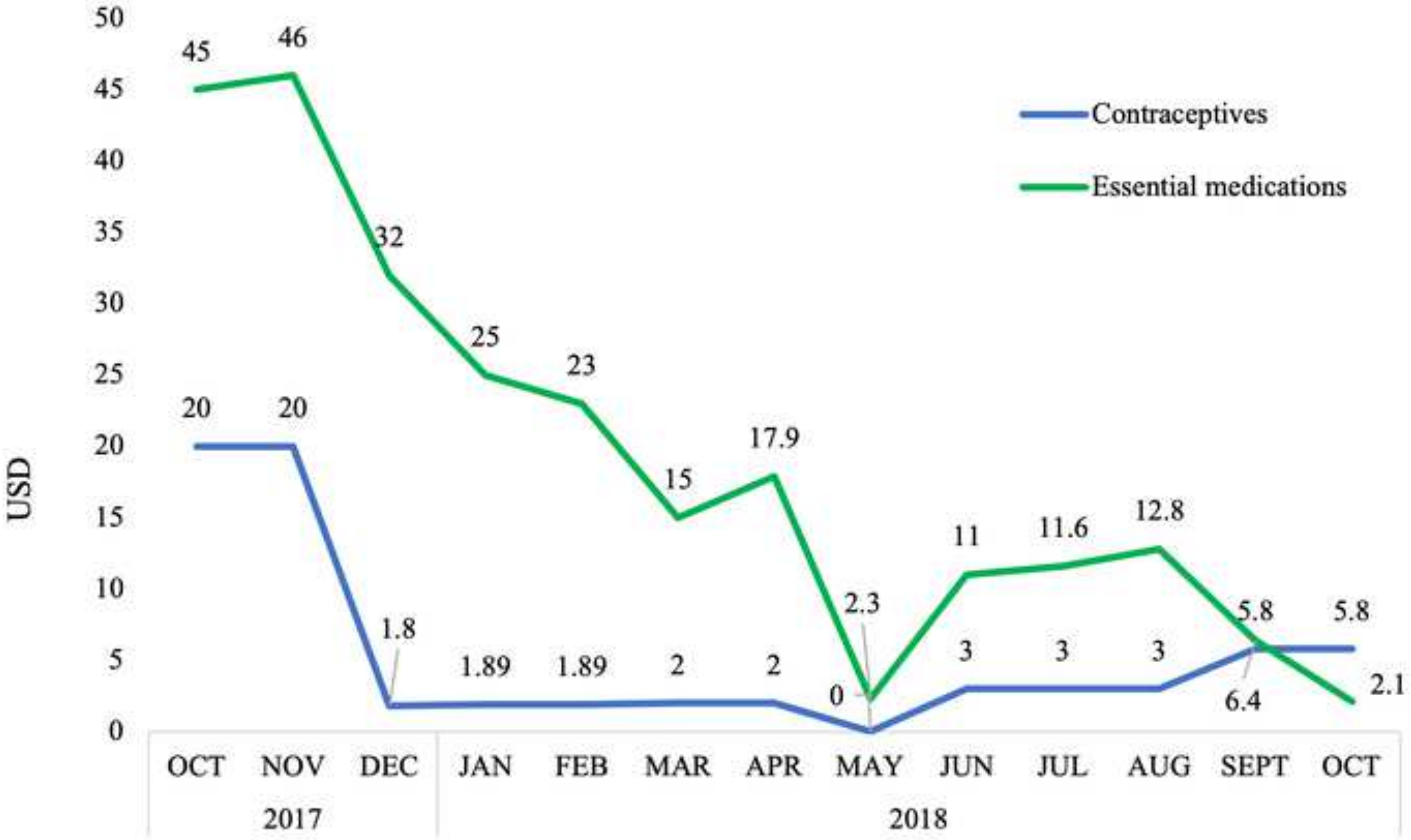

Click here to access/download;Figure;Fig 4.tif

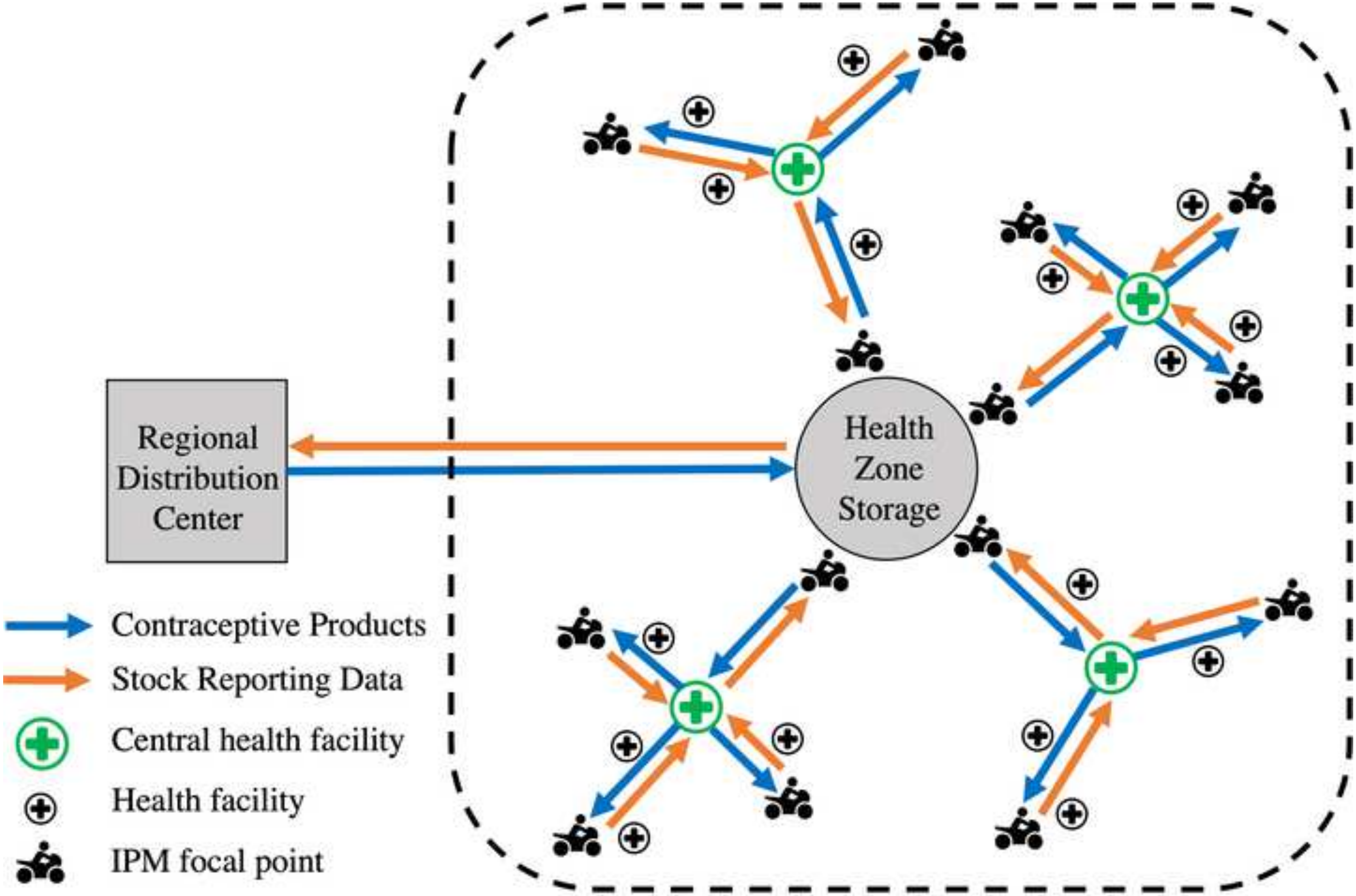

Supplement: Attachment — Submitted filename: PGPH-D-23-01903_reviewer (DRC contraceptives).pdf [file pgph.0003531.s002.pdf]
