# Supplementary material for: Adapting the informed push model to the last mile of the contraceptive supply chain in South Kivu in the Democratic Republic of Congo
Source: PLOS Glob Public Health. 2024 Aug 8;4(8):e0003531. doi: 10.1371/journal.pgph.0003531 (PMC11309402; doi:10.1371/journal.pgph.0003531)
Supplement: Attachment — Submitted filename: Response to Reviewers.docx [file pgph.0003531.s003.docx]

**Authors’ replies to the editors/reviewers’ comments: PGPH-D-23-01903**

**(previous title) Adapting the informed push model to the last mile of the contraceptive supply chain in the Democratic Republic of Congo: Pilot study results**

**(new title) Adapting the informed push model to the last mile of the contraceptive supply chain in South Kivu in the Democratic Republic of Congo**

*Note: in this document, all comments from the editors/reviewers are shown in bold (non-italics). All responses from the authors are shown in non-bold italics.*

*On February 22, 2024 the editor/reviewers sent us a series of new comments to address. Below are those points, as well as our replies.*

**1. Please include a complete copy of PLOS’ questionnaire on inclusivity in global research in your revised manuscript. Our policy for research in this area aims to improve transparency in the reporting of research performed outside of researchers’ own country or community. The policy applies to researchers who have travelled to a different country to conduct research, research with Indigenous populations or their lands, and research on cultural artefacts. The questionnaire can also be requested at the journal’s discretion for any other submissions, even if these conditions are not met.  Please find more information on the policy and a link to download a blank copy of the questionnaire here:** [**https://journal.plos.org/globalpublichealth/s/best-practices-in-research-reporting**](https://journals.plos.org/globalpublichealth/s/best-practices-in-research-reporting)**. Please upload a completed version of your questionnaire as Supporting Information when you resubmit your manuscript.**

*Reply: The questionnaire has been completed and attached as supporting information.*

**2. Please send a completed 'Competing Interests' statement, including any COIs declared by your co-authors. If you have no competing interests to declare, please state "The authors have declared that no competing interests exist". Otherwise please declare all competing interests beginning with the statement "I have read the journal's policy and the authors of this manuscript have the following competing interests:"**

*Reply: The authors have declared that no competing interests exist.*

**Additional Editor Comments:

Reviewers raise several important points about the methods, please read closely reviewer 1 and 5's comments and revise accordingly.**

*Reply: Responses to reviewer 1 and 5’s comments are included below.*

**5. Review Comments to the Author**

**Reviewer #1: This analysis of the informed push model in the DRC contributes to the contraceptive security knowledge base in challenging contexts. The manuscript is, for the most part, well-written. I have included questions and comments on the manuscript.**

*Reply: All spelling and grammar comments that were included in the PDF of the manuscript have been incorporated.*

**I suggest that the authors expand the introduction of the manuscript, including more information related to the challenges of contraceptive security globally.**

*Reply: The introduction has been expanded. Specifically, a new paragraph was added beginning on Line 52 that discusses the two most common supply chain systems for commodity distribution. The following text has been included:*

*There are two common supply chain systems used for commodity distribution, push (allocation) and pull (requisition).(12) Both supply chain systems require effective and efficient management (13); the main difference between the two systems relates to who is making the resupply decisions. In a push system, a higher-level authority, such as health zone manager, calculates the quantity of commodities to push down the chain.(14) In a pull system, lower-level health facilities order commodities as needed, thereby pulling the supplies through the chain.(14)*

*Further, another paragraph was added beginning on Line 58 that discusses the biggest challenges in contraceptive security, with a focus on low- and middle-income countries. The following text was included:*

*Building a strong and efficient supply chain is especially challenging in low- and middle-income countries (LMICs) where resources are limited. Typically, the final link of the supply chain between the health zone (HZ) warehouse and service provider, commonly referred to as the last mile, is the weakest component of the system.(15) A recent systematic literature highlighted several supply chain barriers that contribute to high stockouts of contraceptives in LMICs, particularly in the last mile of the system in rural areas where service providers may be difficult to reach.(7) First, poor communication between different levels in the supply chain results in bottlenecks of commodities due to inadequate information on contraceptive inventory and consumption to inform supply chain decisions, including accurate forecasting of the type and quantity of contraceptives to procure.(7, 16, 17) Second, lack of reliable and well-maintained physical infrastructure, such as road networks and warehouses, as well as insufficient vehicles to navigate rural terrain, were noted as a major barrier to efficient transportation of supplies.(7, 18, 19) Finally, the absence of human resources dedicated to supply chain management leaves the responsibility of maintaining the supply chain to poorly trained staff and overburdened lower-level health workers, resulting in an erratic supply of contraceptives.(7, 16, 19)*

**I also suggest expanding the discussion section of the manuscript, and add a "limitations" section and a "conclusions" section.**

*Reply: The discussion section has been significantly expanded in several ways. First, a paragraph was included with more in-depth discussion of the study results. Beginning on Line 268, the text reads:*

*At baseline, 65.0% of health facilities in Katana had all nine contraceptive methods included in this study (male condoms, female condoms, oral pills (Microgynon and Microlut), injectables (Depo-Provera), IUD, implants (Jadelle and Implanon NXT) and emergency contraceptive pills) available on the day of the survey. Availability of FP in Katana was slightly higher than the national level (50%) reported in the 2017-18 SPA, which was surprising given the rural geography of the HZ.(32) However, several other international organizations conducted FP programs in this area prior to the C-IPM intervention, which may have increased general availability of FP services in Katana. While the timeliness of commodity reports from health facilities submitted to the HZ slightly increased during the study period (from 75.0% to 80.0%), the completeness of reports slightly decreased (from 85.0% to 80.0%). One possible explanation for the slight decline in completeness of reports may be that the head nurse did not finish the report by the reporting deadline, so when the C-IPM focal point came to the health facility to collect the documents the nurse had to submit an incomplete report. The average length of contraceptive stockouts initially increased, from 7 to 12 days. It is unclear why the mean stockout duration initially increased during the study period, this could be the result of challenges in procuring contraceptives further upstream in the supply chain. However, after the first two months of the study, the average duration of contraceptive stockouts decreased to less than two days throughout the remaining 10 months of the study, indicating the C-IPM was responsive to reported stockouts and able to quickly deliver the necessary commodities. Finally, the average transportation cost of delivering contraceptive supplies from the central HZ pharmacy to health facilities decreased by $14.20 US from the beginning to end of the study period, indicating the C-IPM was less expensive system to manage than the original pull system in Katana. After observing the decrease in transportation costs of contraceptives, the HZ authorities requested that other essential medications be included in the supply packages delivered along the C-IPM transportation routes. This request demonstrated that local health officials found the new supply chain model acceptable and suggests they were invested in sustaining the design beyond the study period.*

*Second, another paragraph was added that compares the results from this study to those from the IPM pilot study in Senegal. Beginning on Line 295, the text reads:*

*The results of this study compare to findings from the pilot study that introduced the IPM in Senegal.(19) In both studies, stockouts of all contraceptive methods were quickly reduced in areas where the IPM was implemented and sustained throughout the study period.(19) Additionally, after the pilot study success in Senegal, regional health managers insisted on adapting the IPM to distribute other essential medications, similar to the request made by HZ authorities in the DRC study.(19) Although this addition to the model was not evaluated for cost-effectiveness in Senegal, results from this DRC pilot study show that bundling contraceptives with other essential medications streamlined the delivery process and lowered overall transportation costs in the health zone. A key difference between the Senegal IPM and the C-IPM in the DRC is that the Senegal model had strong support from the national government and was eventually scaled-up to the national level.(19, 26) Although the DRC C-IPM gained strong support from the HZ authorities and the government in South Kivu, buy-in from government leaders in other provinces or national support will be necessary to scale-up the intervention.*

*Third, the limitations and conclusions have been moved to separate sections in the Discussion with subheadings for “Limitations” on Line 309 and “Conclusions” on Line 378.*

**In addition, it may be more logical to move the "lessons learned" section to AFTER the general "discussions" section.**

*Reply: This suggestion has been incorporated in the manuscript. The “Lessons Learned” section has been moved to Lines 329-341.*

**It would be helpful, in your conclusions, to discuss applicability of the informed push model to other challenging contexts.**

*Reply: The conclusions paragraph has been expanded to include a discussion of the applicability of the IPM to a variety of contexts. Specifically, beginning on Line 382, the following text has been included:*

*Implementing a supply chain intervention like the C-IPM may be particularly beneficial for strengthening contraceptive security in contexts where health facilities are overburdened and staff do not have time to submit stock reports to higher management, especially in hard-to-reach areas with limited internet connection that require the use of physical documents. Further, the C-IPM may be especially useful in areas with fluctuating levels of demand for FP. For example, in some regions of the DRC, community-based programs operate by conducting intermittent outreach events connected to health facilities.(34) Thus, the C-IPM may support health facilities to prepare for anticipated increases in commodity consumption due to community events or other demand generating activities, such as FP messaging campaigns. Finally, the CAD model, designed based on lessons learned from the C-IPM pilot study, can alleviate the burden on health facility staff in large rural areas who had to spend considerable travel time picking up the contraceptive products themselves. In sum, the flexibility of the IPM to adapt to varied contexts make this an encouraging health system strengthening intervention to improve contraceptive security through the last mile of the supply chain to ensure all women have reliable access to a range of contraceptive methods.*

**Reviewer #2: Dear Author,

1. The title and abstract of manuscript needs resynthesis as in its current form it is not very informative**

*Reply: The title of the manuscript has been updated to “Adapting the informed push model to the last mile of the contraceptive supply chain in South Kivu in the Democratic Republic of Congo.”*

**2. The methodology needs to be rewritten with appropriate subtitles and omission of word "Pilot" is highly recommended from, methodology section.**

*Reply: The methodology section has been divided into the following subsections: Intervention (Lines 120-177), Study Design (Lines 179-185), Data Sources (Lines 187-201), Key Measures & Analysis (Lines 203-291), and Ethics Statement (Lines 221-230).*

**3. Discussion needs to be elaborate including more references.**

*Reply: The discussion section has been significantly expanded in several ways. First, a paragraph was included with more in-depth discussion of the study results. Beginning on Line 268, the text reads:*

*At baseline, 65.0% of health facilities in Katana had all nine contraceptive methods included in this study (male condoms, female condoms, oral pills (Microgynon and Microlut), injectables (Depo-Provera), IUD, implants (Jadelle and Implanon NXT) and emergency contraceptive pills) available on the day of the survey. Availability of FP in Katana was slightly higher than the national level (50%) reported in the 2017-18 SPA, which was surprising given the rural geography of the HZ.(32) However, several other international organizations conducted FP programs in this area prior to the C-IPM intervention, which may have increased general availability of FP services in Katana. While the timeliness of commodity reports from health facilities submitted to the HZ slightly increased during the study period (from 75.0% to 80.0%), the completeness of reports slightly decreased (from 85.0% to 80.0%). One possible explanation for the slight decline in completeness of reports may be that the head nurse did not finish the report by the reporting deadline, so when the C-IPM focal point came to the health facility to collect the documents the nurse had to submit an incomplete report. The average length of contraceptive stockouts initially increased, from 7 to 12 days. It is unclear why the mean stockout duration initially increased during the study period, this could be the result of challenges in procuring contraceptives further upstream in the supply chain. However, after the first two months of the study, the average duration of contraceptive stockouts decreased to less than two days throughout the remaining 10 months of the study, indicating the C-IPM was responsive to reported stockouts and able to quickly deliver the necessary commodities. Finally, the average transportation cost of delivering contraceptive supplies from the central HZ pharmacy to health facilities decreased by $14.20 US from the beginning to end of the study period, indicating the C-IPM was less expensive system to manage than the original pull system in Katana. After observing the decrease in transportation costs of contraceptives, the HZ authorities requested that other essential medications be included in the supply packages delivered along the C-IPM transportation routes. This request demonstrated that local health officials found the new supply chain model acceptable and suggests they were invested in sustaining the design beyond the study period.*

*Second, another paragraph was added that compares the results from this study to those from the IPM pilot study in Senegal. Beginning on Line 295, the text reads:*

*The results of this study compare to findings from the pilot study that introduced the IPM in Senegal.(19) In both studies, stockouts of all contraceptive methods were quickly reduced in areas where the IPM was implemented and sustained throughout the study period.(19) Additionally, after the pilot study success in Senegal, regional health managers insisted on adapting the IPM to distribute other essential medications, similar to the request made by HZ authorities in the DRC study.(19) Although this addition to the model was not evaluated for cost-effectiveness in Senegal, results from this DRC pilot study show that bundling contraceptives with other essential medications streamlined the delivery process and lowered overall transportation costs in the health zone. A key difference between the Senegal IPM and the C-IPM in the DRC is that the Senegal model had strong support from the national government and was eventually scaled-up to the national level.(19, 26) Although the DRC C-IPM gained strong support from the HZ authorities and the government in South Kivu, buy-in from government leaders in other provinces or national support will be necessary to scale-up the intervention.*

**Reviewer #3: It's a nice work, however, I have some comments:

1. I would suggest to revise the title as " :A pilot study results"**

*Reply: Based on feedback from other reviewers as well, we have modified the title to read “Adapting the informed push model to the last mile of the contraceptive supply chain in South Kivu in the Democratic Republic of Congo.”*

**2. Please make the discussion section more clearer for the reader. Actually, home message must be clear and understandable for readers.**

*Reply: The discussion section has been significantly expanded in several ways. First, a paragraph was included with more in-depth discussion of the study results. Beginning on Line 268, the text reads:*

*At baseline, 65.0% of health facilities in Katana had all nine contraceptive methods included in this study (male condoms, female condoms, oral pills (Microgynon and Microlut), injectables (Depo-Provera), IUD, implants (Jadelle and Implanon NXT) and emergency contraceptive pills) available on the day of the survey. Availability of FP in Katana was slightly higher than the national level (50%) reported in the 2017-18 SPA, which was surprising given the rural geography of the HZ.(32) However, several other international organizations conducted FP programs in this area prior to the C-IPM intervention, which may have increased general availability of FP services in Katana. While the timeliness of commodity reports from health facilities submitted to the HZ slightly increased during the study period (from 75.0% to 80.0%), the completeness of reports slightly decreased (from 85.0% to 80.0%). One possible explanation for the slight decline in completeness of reports may be that the head nurse did not finish the report by the reporting deadline, so when the C-IPM focal point came to the health facility to collect the documents the nurse had to submit an incomplete report. The average length of contraceptive stockouts initially increased, from 7 to 12 days. It is unclear why the mean stockout duration initially increased during the study period, this could be the result of challenges in procuring contraceptives further upstream in the supply chain. However, after the first two months of the study, the average duration of contraceptive stockouts decreased to less than two days throughout the remaining 10 months of the study, indicating the C-IPM was responsive to reported stockouts and able to quickly deliver the necessary commodities. Finally, the average transportation cost of delivering contraceptive supplies from the central HZ pharmacy to health facilities decreased by $14.20 US from the beginning to end of the study period, indicating the C-IPM was less expensive system to manage than the original pull system in Katana. After observing the decrease in transportation costs of contraceptives, the HZ authorities requested that other essential medications be included in the supply packages delivered along the C-IPM transportation routes. This request demonstrated that local health officials found the new supply chain model acceptable and suggests they were invested in sustaining the design beyond the study period.*

*Second, another paragraph was added that compares the results from this study to those from the IPM pilot study in Senegal. Beginning on Line 295, the text reads:*

*The results of this study compare to findings from the pilot study that introduced the IPM in Senegal.(19) In both studies, stockouts of all contraceptive methods were quickly reduced in areas where the IPM was implemented and sustained throughout the study period.(19) Additionally, after the pilot study success in Senegal, regional health managers insisted on adapting the IPM to distribute other essential medications, similar to the request made by HZ authorities in the DRC study.(19) Although this addition to the model was not evaluated for cost-effectiveness in Senegal, results from this DRC pilot study show that bundling contraceptives with other essential medications streamlined the delivery process and lowered overall transportation costs in the health zone. A key difference between the Senegal IPM and the C-IPM in the DRC is that the Senegal model had strong support from the national government and was eventually scaled-up to the national level.(19, 26) Although the DRC C-IPM gained strong support from the HZ authorities and the government in South Kivu, buy-in from government leaders in other provinces or national support will be necessary to scale-up the intervention.*

*Finally, a “Conclusions” section was added after the Discussion which provides key take-away messages on the applicability of the IPM to other challenging contexts. The following text was included on Lines 379-396:*

*Overall, given the large land area of the DRC and poor national infrastructure, building a strong supply chain is challenging, however, the pilot study results indicate it is feasible to adapt the IPM to a new location with complex geographical, political and socioeconomical influences. Implementing a supply chain intervention like the C-IPM may be particularly beneficial for strengthening contraceptive security in contexts where health facilities are overburdened and staff do not have time to submit stock reports to higher management, especially in hard-to-reach areas with limited internet connection that require the use of physical documents. Further, the C-IPM may be especially useful in areas with fluctuating levels of demand for FP. For example, in some regions of the DRC, community-based programs operate by conducting intermittent outreach events connected to health facilities.(34) Thus, the C-IPM may support health facilities to prepare for anticipated increases in commodity consumption due to community events or other demand generating activities, such as FP messaging campaigns. Finally, the CAD model, designed based on lessons learned from the C-IPM pilot study, can alleviate the burden on health facility staff in large rural areas who had to spend considerable travel time picking up the contraceptive products themselves. In sum, the flexibility of the IPM to adapt to varied contexts make this an encouraging health system strengthening intervention to improve contraceptive security through the last mile of the supply chain to ensure all women have reliable access to a range of contraceptive methods.*

**3. Refences are not well cited and maintained any specific style as per PGH guidelines. So, revise them carefully.**

*Reply: The references have been updated according to the PGH guidelines using the Vancouver reference style.*

**Reviewer #4: This article is clearly written and provides useful programmatic information about a promising approach to improving the FP supply chain.

It's interesting that the SPA survey found a 50% stockout rate nationally, but this study found 65% of facilities in the pilot study had all methods, even though this study was conducted in a remote area that might be expected to be worse that the country overall. Authors may want to comment on that in the discussion.**

*Reply: We agree this was an interesting finding. We hypothesize that this result may be due to several years of FP programming provided by other international donors, including the International Rescue Committee and Cordaid, prior to the IPM intervention. Although the funding for these projects was not occurring at the same time as the IPM study, we have included the following text in the discussion section on Lines 271-275:*

*Availability of FP in Katana was slightly higher than the national level (50%) reported in the 2017-18 SPA, which was surprising given the rural geography of the HZ.(32) However, several other international organizations conducted FP programs in this area prior to the C-IPM intervention, which may have increased general availability of FP services in Katana.*

**Minor notes:
Line 163 – I think it should say “reports” instead of reported**

*Reply: This has been revised.*

**Line 195 - CODESA acronym should be spelled out on first reference.**

*Reply: We have included the full name of the Comité de Développement Sanitaire (CODESA) as well as a more detailed description of the community-run group in the first reference in manuscript, now appearing on Line 133.*

**Reviewer #5: Thank you for giving me the opportunity to review this paper. I found the topic very interesting and the study does contribute to filling a gap in drug delivery in resource-poor settings.

METHODS
Pilot Intervention
- Add timing of when the intervention started.**

*Reply: The intervention began at the start of the study period in October 2017. We have included this information on Line 181 in the Study Design subsection in the Methods.*

**- How were the focal points identified from the community?**

*Reply: Focal points were identified from within a roster of active community health workers operating in Katana who were members of the CODESA. The CODESA selected the focal points themselves from within their group. To clarify this point in the manuscript, the following text has been included on Lines 132 – 140:*

*Next, each axis in the model was assigned a C-IPM focal point by the existing HZ management structure, the Comité de Développement Sanitaire (Sanitary Development Committee), or CODESA, which is a community-led committee that manages the primary health care facilities in each HZ and leads health development in their area.(31) Each HZ in South Kivu is managed by a CODESA that consists of community health workers, known locally as Relais Communautaires (RECOs), who were elected to the committee, as described in detail elsewhere.(31) The C-IPM focal points were appointed by the CODESA in Katana from within their list of committee members and were required to be able to read and write in the local language, have a good reputation in the community, and be willing to volunteer for the position.*

**- It’s a bit difficult to understand what drugs the focal points initially started supplying at the beginning of the study and tie that with what was said in other parts of the manuscript.**

*Reply: In months 1-4 of the pilot study, the focal points were only delivering contraceptive commodities to health facilities. Then, in months 5-12 of the pilot study, essential medications for other vertical health programs in the health zone (HIV, tuberculosis, and malaria) were included in the same bundle as the contraceptives and delivered as one package to each health facility by the focal points. To clarify this point in the manuscript, the following text has been included on Lines 169-177:*

*Initially, only contraceptive commodities were distributed in the Katana HZ using the C-IPM. There were nine contraceptive methods included in the IPM, male condoms, female condoms, oral pills (Microgynon and Microlut), injectables (Depo-Provera), IUD, implants (Jadelle and Implanon NXT) and emergency contraceptive pills. However, four months after the pilot study began, the HZ management in Katana decided to expand the C-IPM to include essential medications for the treatment of malaria, tuberculosis, and HIV to maximize transportation efficiency and minimize overall delivery costs in the HZ. Under this change, the HZ pharmacists then included the expanded list of essential medications in the contraceptive bundles to be delivered as one package to the health facilities.*

**Did the monthly commodity consumptions being described in this section include all drugs at the health facilities or only essential drugs or only family planning items?**

*Reply: Information on commodity consumption included all medications at the health facilities. The monthly stock reports in this study were the standard reports used by the health zone management. After the focal points picked up the stock reports, they submitted the documents to the health zone pharmacists who reviewed the reports and uploaded the information to the LMIS system. The monthly stock data used for the analysis in this study was downloaded directly from the LMIS system. This point has been clarified in the manuscript by adding a subsection under Methods for “Data Sources.” The following text was included on Lines 192-199:*

*Second, monthly stock reports that contained information on daily commodity consumption, remaining stock levels, commodities received, and expired products were collected from health facilities by the C-IPM focal points throughout the study period and submitted to the HZ pharmacist. Following the monthly Monitoring Meeting, as described above, information from the stock reports were uploaded to the Logistic Management Information System (LMIS) in the DRC, along with measures for timeliness and completeness of the stock reports. For this analysis, data regarding the monthly stock reports was downloaded directly from the LMIS.*

**In the Pilot Implementation and assessment section, stock reports were collected by the focal points (which is understandable) but then in the results, you describe how health facility staff and HZ management requested to include other essential drugs to the supply list. If my understanding is correct, the contraceptives were meant to be added to the essential medicines list identified for supply. So were these essential drugs not part of the supply list at the beginning of the study? It will be beneficial if you clearly explain what information (in terms of stocks/supplies) was collected at the beginning of the study and how this list changed over time, referring to the HZ management request to add other essential drugs.**

*Reply: The monthly stock reports submitted by the focal points to the HZ pharmacist were the standard reports used by the Katana Health Zone to monitor all medications provided in health facilities, not just contraceptives. However, in this study of the C-IPM, the focal points only delivered contraceptive commodities in months 1-4 of the study, then, the HZ management of Katana made the decision to include medications for HIV, tuberculosis, and malaria in the same bundle as the contraceptives to be delivered as one package to the health facilities by the focal points. As detailed in the responses above, these points have been included in the revised manuscript.*

**Pilot implementation and assessment.
- Line 50: Please specify what you considered to be the key pilot indicators for the study.**

*Reply: The key measures in this study were availability of contraception, duration of contraceptive stockouts, delivery time, transportation cost, timeliness of reports, and completeness of reports. Detailed descriptions of these measures have been included in a new subsection of the Methods; the following text has been included on Lines 204-215:*

*First,* availability of contraception *was defined as a binary variable coded to 1 if there were no stockouts of any of the nine contraceptive methods included in this study at the health facility on the day of the survey. A stockout was defined as one or more methods of contraception unavailable or expired on the day of the survey. Next, the* duration of contraceptive stockouts *and* delivery time *were continuous variables measured in days. The* transportation cost *of delivering the commodities from the HZ pharmacy to the health facilities was a continuous variable measured in USD. Finally, two binary variables were used to measure the quality of the monthly reports submitted by the C-IPM focal points. The* timeliness of reports *was coded to 0 if the report was submitted one or more days after the agreed upon deadline and 1 if the report was submitted to the HZ management by the deadline. The* completeness of reports *was coded to 0 if any information on the report was missing and 1 if all information was included in the report.*

**- How did you define and assess timeliness of stock reports?**

*Reply: Timeliness of stock reports referred to whether the head nurse at each health facility gave the stock report to the focal point on the day the focal point came to collect the documents. The date of when reports were collected was determined by the focal point and health zone management for each distribution axis of the IPM, but all dates were within the first 10 days of each month. This information is included on Lines 156-169.*

**- Who did the focal points submit their reports to?**

*Reply: The focal points submitted the reports to the HZ pharmacist. This information is provided on Lines 159-161.*

**- I imagine the focal points were not necessarily medical professionals or pharmacists. So were there instances where mistakes in stock supplies occurred? And how were this supply mistakes dealt with if these did occur?**

*Reply: The focal points were community health workers who lived and worked in Katana and were members of the CODESA. The HZ pharmacists reviewed all stock reports and order requisitions for accuracy. Any mistakes or issues in the reports were discussed and corrected at the monthly Monitoring Meeting before the data was uploaded to the LMIS. To clarify this point in the manuscript, the following text has been included on Lines 159-165:*

*The focal point would then submit the documents to the HZ pharmacist who reviewed the monthly stock reports and order requisitions for accuracy and packaged the requested contraceptives into bundles for each distribution axis. If the HZ pharmacists noted issues in the reports, these were discussed with the head nurse of the facility at the monthly Monitoring Meeting, which is an institutionalized component of the health system in Katana where health facility staff and HZ management gather to review and validate all data related to the health system.*

**Ethics statement
- I am curious to know if attempts to obtain ethics approval from the national DRC ethics committee (National Committee of Health Ethics/ Comité National d’Éthique de la Santé) was sought for this study. And if not, why? Or were you already given a waiver by the DRC ethics committee?**

*Reply: The study team did not attempt to obtain ethical approval from the national ethics committee (usually the Kinshasa School of Public Health Ethics Committee) after a discussion with the Provincial Department of Health (DPS) in South Kivu. The DPS provided permission for the IPM study to operate in Katana and responded verbally that approval from the local ethics committee was not necessary given that no client data was involved in the study. All indicators used to assess the C-IPM were related to commodities and reporting schedule and did not involve human behavior.* **Pilot results
- Line 163: change ‘reported’ to ‘reports’.**

*Reply: This wording has been revised in the manuscript.*

**- For the reports described in the results section, I propose to specify what reports you are referring to; would that be the reports of monthly commodity consumption that the focal person collects from the nurse or is it the monthly reports that the focal person submits to track the duration of stockouts?**

*Reply: To clarify the different types of reports and data used in this study, a new subsection on “Data Sources” has been added to the Methods section. The following information is provided on Lines 188-201:*

*There were three main sources of data for this analysis. First, baseline and endline surveys of the health facilities in Katana were conducted in-person by local staff in October 2017 and 2018. The surveys included information on contraceptive availability, which was verified by observation by the local researcher, and stockouts, including the occurrence and duration of stockouts by method as reported in the health facility register. Second, monthly stock reports that contained information on daily commodity consumption, remaining stock levels, commodities received, and expired products were collected from health facilities by the C-IPM focal points throughout the study period and submitted to the HZ pharmacist. Following the monthly Monitoring Meeting, as described above, information from the stock reports were uploaded to the Logistic Management Information System (LMIS) in the DRC, along with measures for timeliness and completeness of the stock reports. For this analysis, data regarding the monthly stock reports was downloaded directly from the LMIS. Finally, the C-IPM focal points submitted cost reports to the health zone management for the transportation cost of delivering the commodity bundles each month.*

**- How was completeness of the reports measured? What parameters were used? I suggest this explanation be added to the methods section.**

*Reply: The completeness of reports is a measure determined by the HZ management when the stock reports are uploaded to the LMIS. This is a standard measure included in the DRC’s LMIS system.*

**- And what could have explained the decrease in completeness of reports from baseline to endline?**

*Reply: We hypothesize that the marginal decrease in completeness of reports may be due to the study design if the head nurse at a health facility had not finished the stock reports by the time the focal point came to the health facility to collect the documents, so the nurse submitted the reports as is. This information has been added to the Discussion section on Lines 277-280.*

**- Line 164: What was the definition of ‘on time’ for the study? i.e. was this exact to the pre-agreed date? What happened if the report was not on time? Could the focal person come back at a later date if the report you are referring to here is the nurse’s monthly commodity consumption? All this could be explained in the methods section.**

*Reply: Reports were considered ‘on time’ if the head nurse gave the stock report to the focal point on the day the focal point came to collect the documents at the health facility. If the report was not submitted on that date, the head nurse could bring the report to the monthly Monitoring Meeting. The definition of ‘on time’ reports is included in the “Key Measures” subsection of the Methods on Lines 211-213.*

**- A brief description of how the delivery costs were calculated/determined in the methods section will be beneficial.**

*Reply: The delivery costs were determined by how much the focal point paid to hire a local motor taxi to deliver the commodities, which was information recorded in the health facility register and reported monthly by focal points. This information is included on Lines 199-201 in the Methods section.*

**Lessons learned
- Line 194-196: The involvement of CODESA (please define as this is the first time it’s being used in the document) to appoint the focal persons from the community should have been first described in the methods section.**

*Reply: A more in-depth description of the CODESA and how focal points were selected is now described on Lines 132-140 in the Methods section. The text reads as follows:*

*Next, each axis in the model was assigned a C-IPM focal point by the existing HZ management structure, the Comité de Développement Sanitaire (Sanitary Development Committee), or CODESA, which is a community-led committee that manages the primary health care facilities in each HZ and leads health development in their area.(32) Each HZ in South Kivu is managed by a CODESA that consists of community health workers, known locally as Relais Communautaires (RECOs), who were elected to the committee, as described in detail elsewhere.(32) The C-IPM focal points were appointed by the CODESA in Katana from within their list of committee members and were required to be able to read and write in the local language, have a good reputation in the community, and be willing to volunteer for the position.*

**Revised intervention design
- At what time point in the study was the CAD implemented?**

*Reply: The CAD model was not included in the study; it was designed based on lessons learned from the C-IPM study. To clarify this in the manuscript, the description of the CAD model has been moved to a new subsection for “Future Directions” in the Discussion on Lines 344-354.* **Discussion
You may need to critically develop your discussion a bit further. For example, how did your results differ from other contexts that have implemented this IPM? And why? Or how are they similar if this is the case?**

*Reply: The discussion section has been expanded to include a paragraph that compares the C-IPM pilot study to the IPM pilot study in Senegal. The following text has been added on Lines 295-307:*

*The results of this study compare to findings from the pilot study that introduced the IPM in Senegal.(20) In both studies, stockouts of all contraceptive methods were quickly reduced in areas where the IPM was implemented and sustained throughout the study period.(20) Additionally, after the pilot study success in Senegal, regional health managers insisted on adapting the IPM to distribute other essential medications, similar to the request made by HZ authorities in the DRC study.(20) Although this addition to the model was not evaluated for cost-effectiveness in Senegal, results from this DRC pilot study show that bundling contraceptives with other essential medications streamlined the delivery process and lowered overall transportation costs in the health zone. A key difference between the Senegal IPM and the C-IPM in the DRC is that the Senegal model had strong support from the national government and was eventually scaled-up to the national level.(20, 27) Although the DRC C-IPM gained strong support from the HZ authorities and the government in South Kivu, buy-in from government leaders in other provinces or national support will be necessary to scale-up the intervention.*

**Do you believe that the use of the focal points from the communities has markedly improved access to contraceptives (and other essential drugs) compared to other contexts?**

*Reply: The study presented in this manuscript was designed to test the feasibility of adapting the IPM to the context of one health zone in the DRC. Thus, the results do not provide information to compare to other contexts. Throughout the manuscript, we have carefully worded our analysis to “availability of contraceptives” in health facilities as we recognize that access to family planning services may be limited due to other financial, geographic, and social barriers.*

**Did the improvements in the availability of the contraceptives at the health facilities translate to a higher uptake of these products by women?**

*Reply: This study did not include information on women’s contraceptive behaviors; therefore, we cannot determine whether the C-IPM contributed to increased use of modern contraception in Katana. Further, no population-based surveys have occurred in the Katana health zone during or following the C-IPM study.*

**Is the decrease in time of stock-outs a reflection of the success of the IPM (and maybe improved uptake of the contraceptives) or was there the possibility that nurses were now being more conscientious in their stock management knowing that there is the added scrutiny of the study?**

*Reply: We theorize that the decrease in stockout duration was due to increased responsiveness of the C-IPM to shocks in the supply as focal points were able to quickly and efficiently deliver contraceptive commodities to health facilities experiencing a stockout and the head nurse was no longer required to take time away from work to collect the products that were out of stock. However, the review raises an interesting point that the study design may have affected the behavior of head nurses at the health facilities if they were aware of added scrutiny from health zone management during the study. We have included this point in the Limitations section on Lines 314-317.*

**What could explain the sharp increase in the mean length of stock-out days from October to November 2017?**

*Reply: We do not have a clear answer as to why the mean stockout length increased from 7 days in October to 12 days in November, before declining for the remaining study period. However, it is possible there may have been challenges in procurement of contraceptives further upstream in the supply chain. This note has been added on Lines 280-283.*

**Please submit your revised manuscript by Apr 07 2024 11:59PM. If you will need more time than this to complete your revisions, please reply to this message or contact the journal office at globalpubhealth@plos.org. When you're ready to submit your revision, log on to** [**https://www.editorialmanager.com/pgph/**](https://www.editorialmanager.com/pgph/) **and select the 'Submissions Needing Revision' folder to locate your manuscript file.

Please include the following items when submitting your revised manuscript:**

- **A rebuttal letter that responds to each point raised by the editor and reviewer(s). You should upload this letter as a separate file labeled 'Response to Reviewers'.**
- **A marked-up copy of your manuscript that highlights changes made to the original version. You should upload this as a separate file labeled 'Revised Manuscript with Track Changes'.**
- **An unmarked version of your revised paper without tracked changes. You should upload this as a separate file labeled 'Manuscript'.**

**Guidelines for resubmitting your figure files are available below the reviewer comments at the end of this letter.

We look forward to receiving your revised manuscript.**
